# Effectiveness of different sounds in human echolocation in live tests

**DOI:** 10.1371/journal.pone.0306001

**Published:** 2024-10-17

**Authors:** Michał Bujacz, Aleksandra Królak, Bartłomiej Sztyler, Piotr Skulimowski, Paweł Strumiłło

**Affiliations:** Institute of Electronics, Lodz University of Technology, Lodz, Poland; Chunghwa Telecom Co. Ltd., TAIWAN

## Abstract

Echolocation is a vital method of spatial orientation for many visually impaired individuals who are willing to and able to learn it. Blind echolocators use a variety of sounds, such as mouth clicks, cane taps, or specialized sound-emitting devices, to perceive their surroundings. In our study, we examined the effectiveness of several different sounds used in echolocation by conducting trials with 12 blind and 14 sighted volunteers. None of the participants had received formal training in echolocation, though a number identified as self-taught experts. The sounds tested included those played from a loudspeaker, generated by a mechanical clicker, or made by the participants themselves. The task given to the participants was to identify the direction and distance to an obstacle measuring 1x2 meters in an outdoor environment, with the obstacle placed in one of nine possible positions. Our findings indicated that the blind participants displayed significantly better echolocation skills when compared to the sighted participants. The results of the blind participants were also strongly divided into two distinct subgroups—totally blind participants performed much better than those which were legally blind, but had some residual vision. In terms of sound comparisons, we found that sounds with a center frequency near 3-4kHz and a wide spectrum provided higher accuracy rates than those with lower frequency peaks. Sighted participants performed best with 3kHz and 4kHz percussion sounds, while the blind group performed best with blue and pink noise. The loudspeaker generated tones generally yielded better results than those generated by the participant (using a mechanical clicker, mouth clicks or hand claps). These results may be useful in developing training programs that teach echolocation as well as artificial sounds to improve echolocation effectiveness.

## 1. Introduction

Echolocation is the ability to perceive the environment using reflected sounds. It is common knowledge that some animals such as bats and dolphins use this ability naturally [1], but it is a lesser-known fact that all humans also can “unlock” and train this skill [2]. In the blind community, there are famous expert echolocators who can echolocate even while riding bikes [3], there are courses to teach echolocation to blind children and adults [4, 5], and there is a growing body of research on various aspects of this phenomenon, from mouth clicks [6] to brain activity imaging of expert echolocators [7]. This paper aims to contribute to the studies of human echolocation by analyzing the influence of various sound types that could be used to echolocate on the accuracy of echolocation by both blind and sighted individuals.

Common types of sounds that can be used as the source signal for echolocation are tongue clicks, hisses [8], hand claps [9], finger snaps [10] or even naturally occurring sounds of footsteps or cane taps [3]. Mechanical metal clickers or wooden castanets can also be used. The studies of the sounds used in echolocation were mainly carried out by Rojas [10, 11], Smith [12] and Thaler [13, 14]. They investigated various hand, mouth, and artificially generated sounds.

The presented study investigated the effectiveness of different sounds used in echolocation in natural conditions. Ten sounds were used in tests conducted by 12 blind and 14 sighted volunteers with the goal to identify the location of a large obstacle at ranges from 1 to 3 meters.

### State of the art

There is no set consensus on the most effective sound sources for echolocation, and very few studies have shown strong significant advantages of a single sound type. This may be due to the strong individual differences between echolocators [15], majority of studies being conducted on very small groups of participants [16], differing methodologies and studies usually focusing on comparison of just one or two parameters or types of sounds [17]. However, the general agreement is that sounds suitable for echolocation should have a peak frequency in the 1.5–4.5 kHz range [12] and a wide spectrum containing some energy in the high frequency range [3]. The peak frequency corresponds to the region of highest sensitivity of human hearing [18], while the influence of the content of higher frequency components may be due to the mechanisms of sound localization, especially the head-related transfer function’s largest variations in the upper part of the spectrum [19].

A recent study by Norman and Thaler [20] suggests that the high frequency content may be important not due to the properties of human hearing, but primarily because of the reflectivity of objects used in echolocation studies, as echolocation tests with various source frequencies, but with echoes artificially equalized across the spectrum lead to similarly accurate results. It is true that the reflectivity characteristics of various surfaces have a strong influence on echolocation [21].

Commonly used echolocation sounds can be divided into three main categories: hand-generated (claps or snaps), mouth-generated (e.g. palatal clicks), and various artificial sounds, either generated mechanically or synthesized and played back from speakers (e.g. artificial clicks, transients, or noises) [17].

Several papers by Prof. Rojas analyzed different mouth- generated sounds, such as the most common palatal clicks, hissing sounds [8], oral and lip ’ch’ sound, an ’iu’ sound, and whistling [11] as well as hand-generated sounds, like finger snapping and clapping [10]. Results comparing the repeatability and intensity of the sounds (though not their practical effectiveness) suggest that the oral-produced click is the most suitable for human echolocation. From hand-generated sounds the little known “knuckle vacuum pulse” (slapping a finger on the space between the knuckles of a closed fist) seems to be a strong candidate for a good echolocation sound, due to its high frequency and similarity to palatal clicks [10].

A highly cited study by Thaler et al. [14] attempted to analyze what made palatal clicks of expert echolocators different from those of novices led to the conclusion that the experts’ clicks were very consistent, short (3ms), have a wide energy band (with high energy content in up to 10kHz), and a peak frequency in the range of 2-4kHz. A synthetic model of those clicks was utilized in a number of echolocation studies, including this one.

There is some discussion about whether sounds reproduced from a loudspeaker or those generated by the echolocators themselves are more effective for echolocation. Results suggest that in various scenarios, artificial clicks are comparable or slightly better than real clicks [6, 22], especially for novice sighted echolocators [23]. One explanation for this may be the perfect repeatability of artificial signals. Studies showed that familiarity with a sound’s spectrum and its conformance to expectations play a significant role in echolocation accuracy [24].

Among sounds other than clicks, noise, usually white, is often utilized in echolocation studies; however, there is no clear conclusion as to its effectiveness. In one study, white noise was better than clicks [25], in another [26] worse, or there was no statistically significant difference [24].

One interesting study [27] used bursts of click-trains to see what frequency of signal repetition resulted in highest echolocation accuracy. The conclusion was that for nearby obstacles (up to 1m) the higher repetition rates resulted in improved obstacle detection with continuous white noise producing the best results, while at larger distances the optimum repetition was every roughly 15ms (the study used 64 noise bursts per second, each lasting 5ms).

Echolocation testing scenarios can vary from experiments with recordings or synthesized sounds [28], virtual audio, various controlled laboratory environments to real-life settings, each with their own advantages and limitations [17].

While the use of recordings or synthesized sounds allow for highly controlled and repeatable tests [29–32] trials in real-life or controlled settings [33] may offer a more accurate representation of the typical challenges faced by blind individuals who rely on echolocation for navigation. In real-world environments, echolocators encounter a diverse range of objects, surfaces, and acoustic properties, allowing them to develop and refine their skills in complex and dynamic situations [4].

Studies conducted in real-world settings enable the observation of adaptive strategies employed by blind individuals [33, 34], such as adjusting their emitted clicks [35, 36] or body movements to optimize the received information [37]. Additionally, real-life settings expose echolocators to various background noises, which are crucial for understanding the robustness of echolocation under less-than-ideal conditions. An interesting observation made by experienced echolocators who participated in the authors’ previous studies was the role of the “acoustic shadow”–the echolocators would focus not only on where the first reflection was coming from, but where further echoes from the environment were blocked by an obstacle [38].

## 2. Materials & methods

### Participant group

The echolocation trials were conducted with 12 legally blind and 14 normally sighted (S) volunteers aged between 20 and 65 years of whom there were 12 women and 14 men. Originally, the group was to have 13 blind participants and equal division of sexes, but there was one last moment replacement. Unless otherwise specified, the term “blind” will be used to encompass all legally blind participants, including those with Visual Impairment (VI) and those who are Totally Blind (TB). This division was also not initially planned, but during testing the differences between these subgroups were so large they could not be ignored. None of the blind participants received formal echolocation training, but two participants, TB5 and TB7, referred to themselves as “self-taught echolocation experts” which was clearly visible in their performance.

Participant details are presented in Table 1. The participants were all volunteers recruited between 01.10.2020 and 31.10.2021 from the local Union of the Blind, previous research projects, university students and/or staff. They all signed participant consent forms and received a small remuneration upon completion of all the tests. The study was not double-blind, i.e. the authors could identify all participants and their individually collected data. Anonymization was only performed for publication purposes.

**Table 1:** Participants taking part in echolocation trials.

Participant ID	Age (sex)	Age at loss of sight	Level of vision impairment*	Audiogram results (ear and frequency of worst loss)
TB1	20 (M)	0	Totally Blind	Healthy hearing
TB2	52 (F)	0	Totally blind	Healthy hearing
TB3	42 (M)	4	Totally Blind	Healthy hearing
TB4	39 (F)	6	Totally Blind	Healthy hearing
TB5	48 (M)	6	Totally Blind**	Mild loss (left and right 4-8kHz)
TB6	27(F)	0	Totally Blind	Healthy hearing
TB7	39 (M)	0	Totally Blind**	Healthy hearing
VI1	46 (F)	24	Visually impaired	Healthy hearing
VI2	52 (M)	19	Visually impaired	Mild loss (left and right 4kHz)
VI3	21 (F)	4	Visually impaired	Healthy hearing
VI4	36 (F)	0	Visually impaired	Healthy hearing
VI5	39 (M)	0	Visually impaired	Mild loss (left 1-8Hz) and,
				major loss (right 1-4kHz)
S1	31 (F)	-	Healthy Eyesight	Healthy hearing
S2	62 (M)	-	Healthy Eyesight	Mild loss (left and right 8kHz)
S3	47(M)	-	Healthy Eyesight	Healthy hearing
S4	65(F)	-	Healthy Eyesight	Major loss (left and right 2kHz)
S5	23(F)	-	Healthy Eyesight	Healthy hearing
S6	43 (M)	-	Healthy Eyesight	Healthy hearing
S7	21(F)	-	Healthy Eyesight	Healthy hearing
S8	65(M)	-	Healthy Eyesight	Major loss (right 4-8kHz)
S9	39 (M)	-	Healthy Eyesight	Healthy hearing
S10	36(F)	-	Healthy Eyesight	Healthy hearing
S11	27(M)	-	Healthy Eyesight	Healthy hearing
S12	33(M)	-	Healthy Eyesight	Healthy hearing
S13	41(M)	-	Healthy Eyesight	Healthy hearing
S14	26(F)	-	Healthy Eyesight	Healthy hearing

*all visually impaired participants were legally blind under Polish law (vision worse than 5/50 and field of vision less than 30%) and blindfolded for the experiments

**these two participants are referred to as echolocation experts throughout the text

Prior to trials, all participants underwent standard audiogram examinations at frequencies: 250Hz, 500Hz,1kHz, 2kHz, 4kHz, 8kHz. Three participants were found to have a major hearing loss (above 40dB) in the 4-8kHz spectrum range, and four participants had a minor hearing loss (20-40dB) in mid and/or high frequencies. However, no statistically significant relation was found between the participants’ audiograms and their performance in the echolocation tests.

### Experimental setup and procedure

The tests consisted of echolocating an obstacle in a real outdoor environment (a large park). The obstacle was a wall 2m high and 1m wide, made from a rigid acoustically reflective foamed polycarbonate sheet, set on a wooden frame.

**Fig 1:**
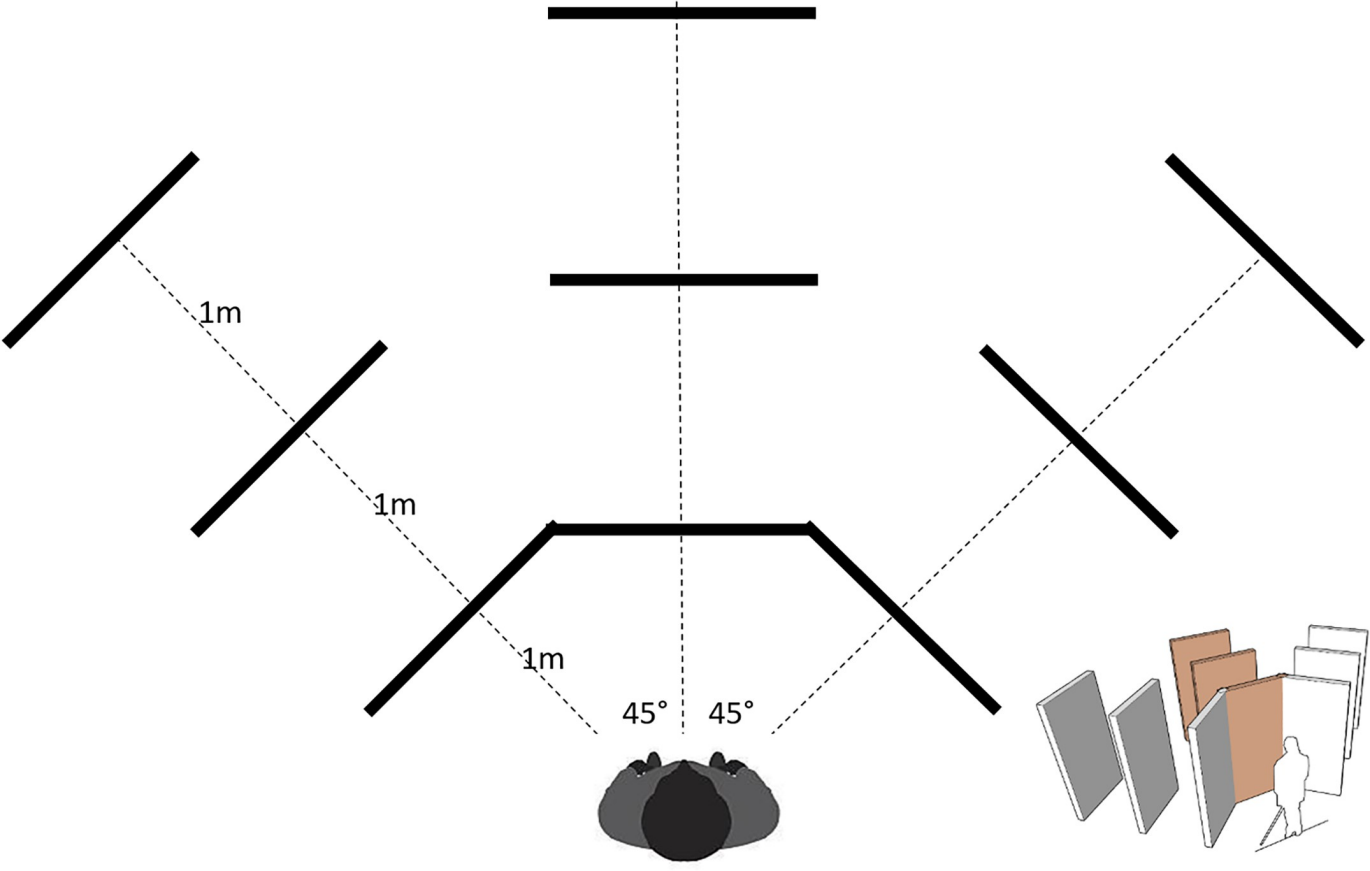
The echolocation experiment setup. The large obstacle was placed in one of 9 possible positions and the participants made a guess about its position, i.e. its direction (left, center, right) and distance (1 m, 2 m, 3 m). The answer was given first after a single echolocation signal (mouth click, hand clap or played back from a waist-height speaker worn on a lanyard) and then after N (up to 10) additional signal repetitions. Each participant went through all 9 locations in a random order in 10 separate sessions using different sounds.

Each of the participants (sighted and visually impaired ones were blindfolded) stood at a fixed place in front of the obstacle. During the tests the obstacle was moved to one of nine pre-determined positions, as illustrated in Fig 1. The obstacle could be placed in front of the participant, at a 45° angle to the left, or at a 45° angle to the right, at a distance of 1m, 2m, or 3m.

The participant’s task was to identify the direction (right, left or centre) and distance (1 meter, 2 metres or 3 metres) to the obstacle by using an echoing sound. The procedure was similar to the ones used in previous tests conducted by the authors [39] as well as other researchers evaluating echolocation [40]. The scenario is a good compromise between efficiency and realism, but is very time consuming, requiring a short pause to quietly move the obstacle.

The emissions of sounds originated either from the participants’ mouths, hands or from a waist-height Bluetooth UE Roll speaker hanging on a lanyard from the participant’s neck. The placement of the speaker was chosen to keep the source sound far away from the face [35]. The loudness of the emissions was adjusted individually by each participant, but the exact SPL was not measured (it was roughly 60-70dB). All participants received the same instruction as to set the loudness of the speaker to a maximum comfortable level and to make their own emissions (mouth clicks or clapping) as loud as possible.

The participant first generated the echolocation signal once, and basing on the echo judged in the direction and distance to the obstacle. This was referred to as the ‘first guess’, then the sound could be repeated by the participant up to 10 times until either they either felt more confident in their answer or decided to change it. The second answer is referred to in the latter text as the answer ‘after N signal repetitions’.

The participant also assessed for confidence in the correctness of their answers on a 5-point scale after the first guess and the second guess after N repetitions. The scale was from 1 –“guessing completely at random”, to 5 –“totally certain, willing to bet on it”.

Once the final answer was given the participant received feedback on the correctness and the obstacle was moved to a different location. While the obstacle was being moved, the participant wore headphones over their ears and listened to music while counting loudly to ten, to prevent overhearing where the obstacle was being placed.

Each participant performed the test in short 9-question series (all possible locations) with each of the 10 sounds in random order. For each sound there were 9 possible obstacle locations presented in a pseudo-random order.

### Tested sounds

The ten sounds used for the experiment were selected basing on the authors’ previous research [38] and other echolocation studies. White noise is frequently used in various psychoacoustic studies, especially as a reference sound [27], but instead it was replaced with pink and blue noise, which allowed to compare the influence of the high and low spectral content. Five percussive sounds were chosen around the frequency human hearing is most susceptible to (~3kHz) [12]. In addition, we also added an exemplary synthesized expert echolocator’s click that has been used in several other studies [14], a mechanical clicker and the participants own preferred sound.

The sounds used to echolocate were played from an Ultimate Ears ROLL 2 speaker (1 kHz, 2 kHz, 3kHz, 4 kHz or 5 kHz drums, pink or blue noise), generated with a mechanical dog-clicker or performed by the test participant without any tools. For the ‘own’ sound category, the majority of blind participants used mouth clicks (with two using hand clapping and one cane tapping), while sighted participants primarily chose hand clapping [9], with two attempting mouth clicks and one using finger snapping.

**Fig 2:**
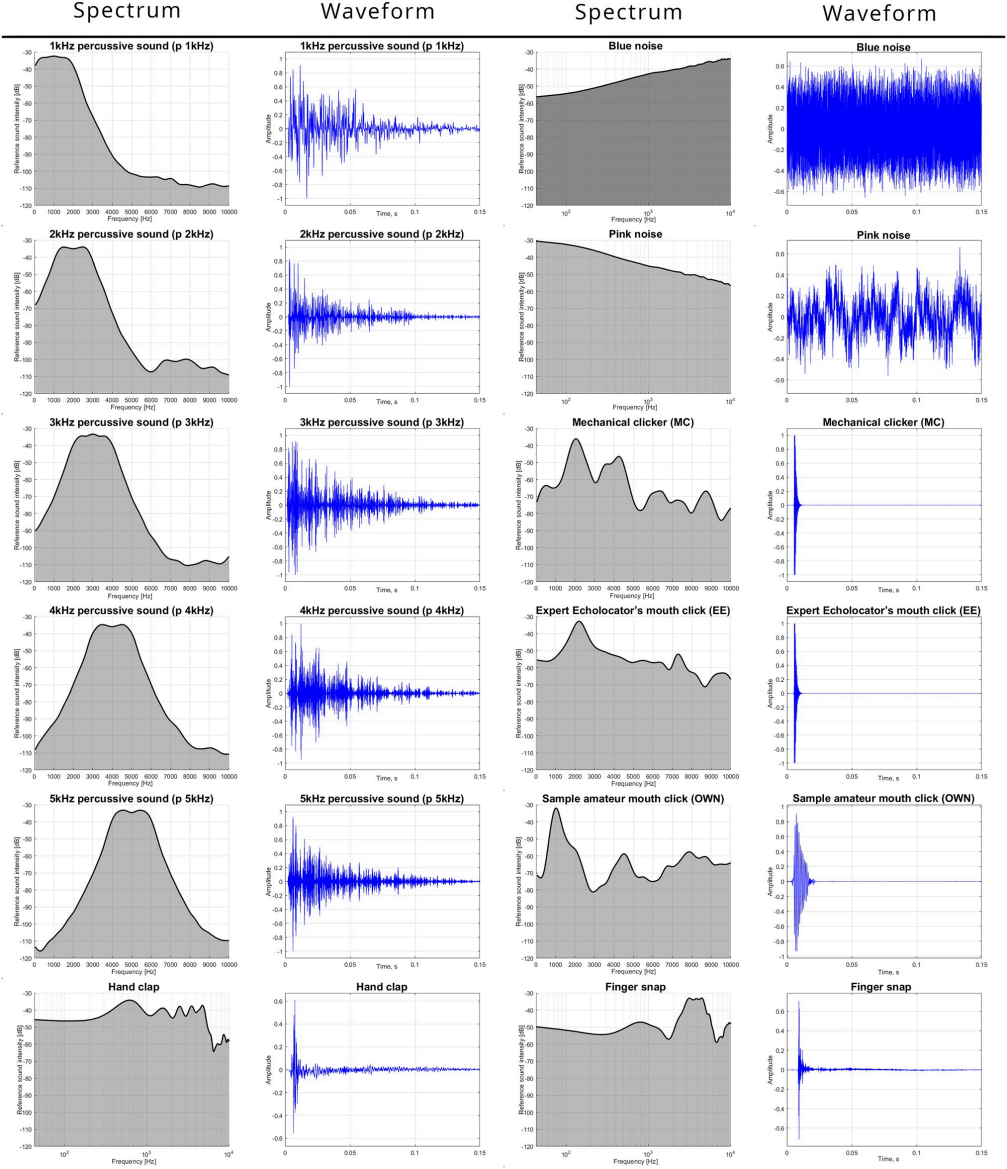
Comparison of sounds used for echolocation.

Plots of the echoing sound signals together with their corresponding Fourier spectra are shown in Fig 2. Note that the frequency axis of each spectrum is given on a logarithmic scale and the vertical axis is scaled in decibels.

The first five sounds (left half of Fig 2) are percussion sounds synthetically generated using the classic Jean Claude Risset’s drum algorithm [41] implemented in the open-source Audacity software [42]. This sound is composed of a band-filtered noise, an enharmonic tone, and a base frequency sine wave [43]. For all the sounds the noise band was set to be 2kHz wide around the center frequency, the noise ratio was 100% and the decay time was set to 0.5s, resulting in a sharp “wooden” percussive sound. The actual audible part of the sound was under 150ms. The use of purely harmonic sounds in the 1-5kHz range was also initially considered, but preliminary tests showed that without the addition of noise the correctness rates were extremely low. Percussive sounds centered 6kHz and above were also eliminated in the preliminary selection due to very low audibility and pleasantness.

The first column of Fig 2 contains spectrograms of the percussive sound sources. Each of them is defined by a specific fundamental frequency. For example, a 3kHz sound means it is a synthetic percussion sound with a dominating frequency of 3khz and 2kHz noise bandwidth (-3dB). Each of the percussion impulses were about 0.1s long.

The next two sounds used in the experiment (the upper two sounds in the right half of Fig 2) were short 250ms noise bursts. We compared blue noise and pink noise. As can be noticed, these noises differ fundamentally in the distribution of power spectral density (PSD). Pink noise is a signal whose power spectral density decreases with increasing frequency. Conversely, for blue noise, the power spectral density increases with frequency. The rate of increase/decrease is about 3dB per octave.

The mechanical clicker used in the tests was a typical device frequently used in dog training. Its main element is a small flexible steel plate. Pressing on the plate bends it, resulting in a characteristic “click” sound. Two distinct peaks in the frequency spectra can be observed in Table 1 (third row and third column). One at 2.1kHz and the other at 4.15kHz. These peaks result from the physical dimensions the rectangular metal plate inside the clicker. The resonance phenomenon can occur both longitudinally and transversely, creating two different frequency modes. The button operated clicker has two distinct clicks, one upon pressing and one upon releasing of the button that deformed the metal plate. However, the release could be significantly delayed and muted if the pressure was maintained on the button as it was slowly eased up. This was how participants were instructed to use the clicker so that they could limit the clicker to a single “main” emission.

The expert echolocator’s mouth click was synthesized using a Matlab script (EE2) provided in the paper by Thaler et al. that analyzed the spectral and temporal characteristics of three expert echolocators [14]. The default settings resulted in a sound with a relatively flat frequency characteristic, with one distinct peak observed at 2.2kHz (see fourth row and third column in Table 1). As with the mechanical clicker, the expert’s click was a very short impulse of about 15ms.

The final tested sound was to be the participant’s own mouth click. However, many subjects (especially the sighted ones) had difficulty in producing consistently loud palatal clicks [44], so the procedure was changed to allow any sound produced by the participants themselves. Most of the blind participants chose mouth clicks, while the sighted participants predominantly clapped [9]. One sighted participant used finger snapping (with much success) and one blind participant tapped her cane on the ground (also with very good results). Sample recordings of these sounds were made using a UMIK1 measurement microphone in a dampened room in front of a BM800 isolation shield, then normalized to the same maximum levels as the synthetic sounds.

## 3. Results

During the echolocation trials, data were collected for 26 participants, each of whom listened to 10 series of sounds, each series consisting of 9 obstacle locations. Every answer was recorded twice—first after hearing the sound emission once, and later after N (up to 10) signal repetitions. Each answer consisted of three parts—the distance, the direction and certainty. When discussing the results “correctness” unless specified otherwise refers to identifying both the direction and distance correctly. The participants also assessed their certainty in each answer twice—after the first and after the last repetition of the signal.

The anonymized raw data are made available at the Lodz University of Technology echolocation project website (direct link at bottom of the page): https://ie.p.lodz.pl/projekty-w-realizacji/2020-2022-echolokacja or from the direct link: https://ie.p.lodz.pl/sites/i23/files/2023-11/outdoor_raw_results_0.xlsx.

### Data statistical analysis

Statistical analysis was performed using mixed ANOVA analysis (Table 2) followed by pairwise comparison with the Mann-Whitney U Test and one-way ANOVA depending on the characteristics of the analyzed data. Before performing the statistical analysis the data was checked for assumptions of sphericity, normality and homogeneity of variances to avoid type II errors. Although originally the study was planned for two groups: sighted vs blind, it became much clearer that three distinct groups emerged:: sighted, visually impaired (legally blind, but with some residual visual perception) and totally blind. The mixed ANOVA (Table 2) was performed separately for the distance and direction accuracy estimation, as well as for certainty values and the final number of N clicks after which the participants settled on their final answer. The data sets were considered statistically different if significance level value α (p-value) was less than 0.05.

**Table 2:** Mixed ANOVA parameters.

	DoF (numerator)	DoF (denumerator)	F-value	α (p-value)	Effect size
**Distance estimation correctness after 1st emission**					
**Group**	2	23	8.538	**0.002**	0.427
**Sound**	9	207	2.259	0.020	0.089
**Group vs. sound**	18	207	1.618	0.058	0.123
**Distance estimation correctness after N emissions**					
**Group**	2	23	3.575	0.044	0.237
**Sound**	9	207	3.244	**0.001**	0.124
**Group vs. sound**	18	207	1.250	0.224	0.098
**Direction estimation correctness after 1st emission**					
**Group**	2	23	5.095	0.015	0.307
**Sound**	9	207	3.752	**<0.001**	0.140
**Group vs. Sound**	18	207	0.950	0.519	0.076
**Direction estimation correctness after N emissions**					
**Group**	2	23	5.724	0.010	0.332
**Sound**	9	207	3.968	**<0.001**	0.147
**Group vs. Sound**	18	207	1.219	0.248	0.096
**Certainty after 1 emission**					
**Group**	2	23	29.420	**<0.001**	0.719
**Sound**	9	207	0.969	0.467	0.040
**Group vs. Sound**	18	207	0.558	0.926	0.046
**Certainty after N emissions**					
**Group**	2	23	24.804	**<0.001**	0.683
**Sound**	9	207	1.101	0.363	0.046
**Group vs. Sound**	18	207	1.320	0.177	0.103
**Number of emissions (N) to finalize answer**					
**Group**	2	23	7.847	**0.003**	0.406
**Sound**	9	207	3.213	**0.001**	0.123
**Group vs. sound**	18	207	2.734	**<0.001**	0.192

**Fig 3:**
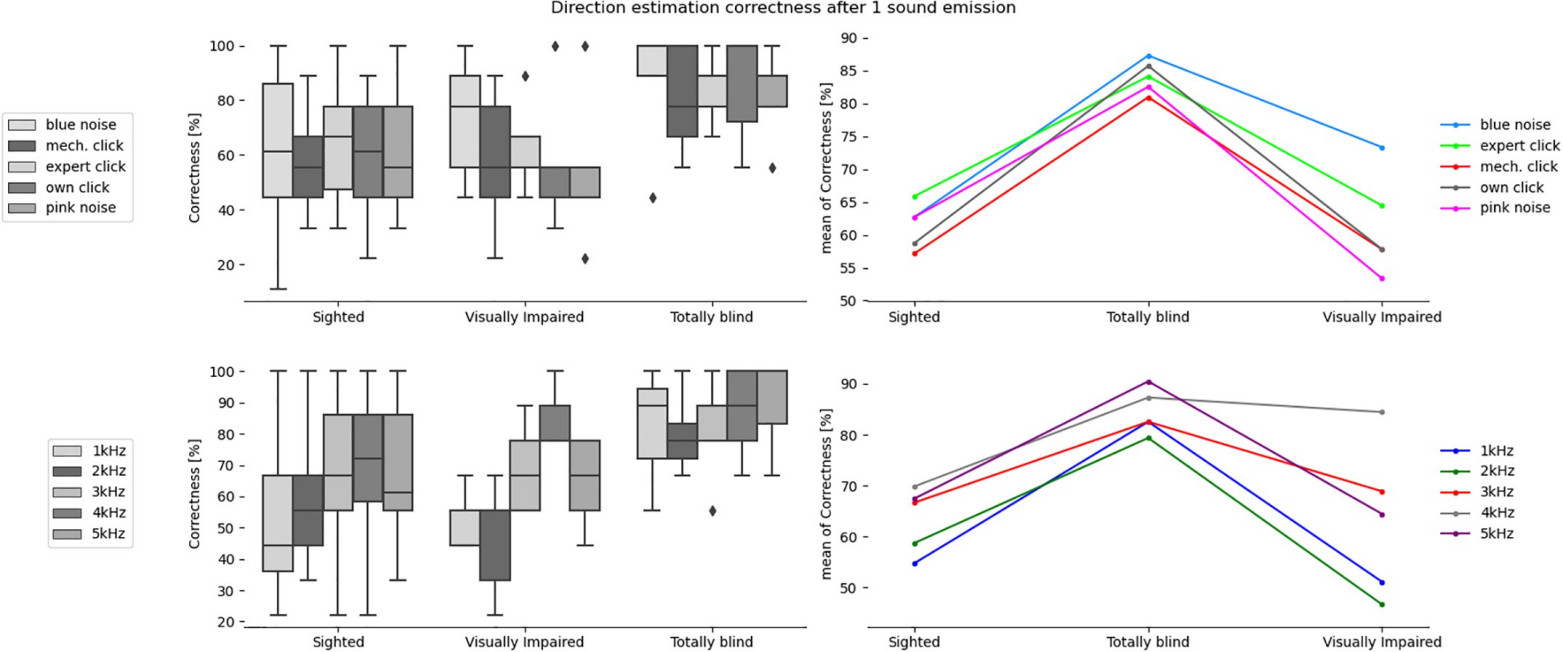
Correctness in estimating direction after a single sound emission for the tested sound types and participants divided into sighted, totally blind and visually impaired groups.

**Fig 4:**
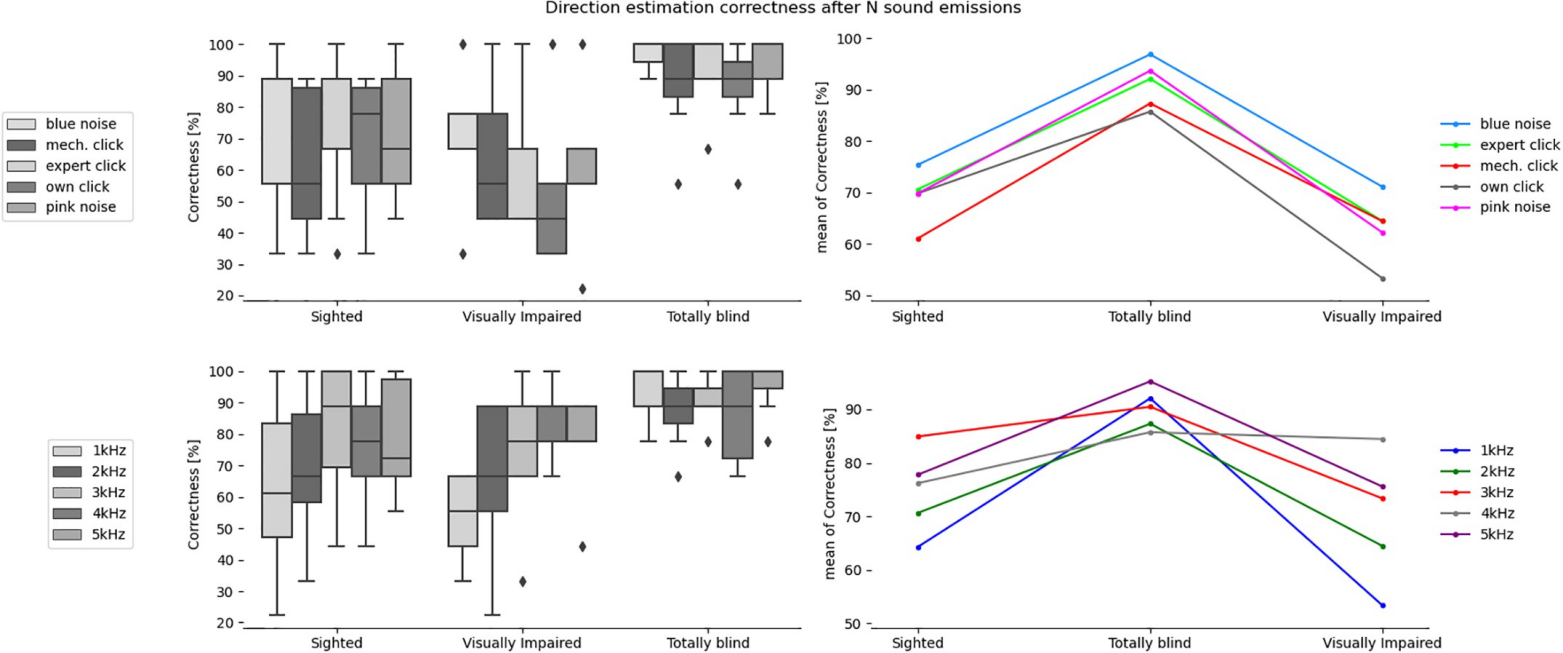
Correctness in estimating direction after N sound emissions for the tested sound types and participants divided into sighted, totally blind and visually impaired groups.

**Fig 5:**
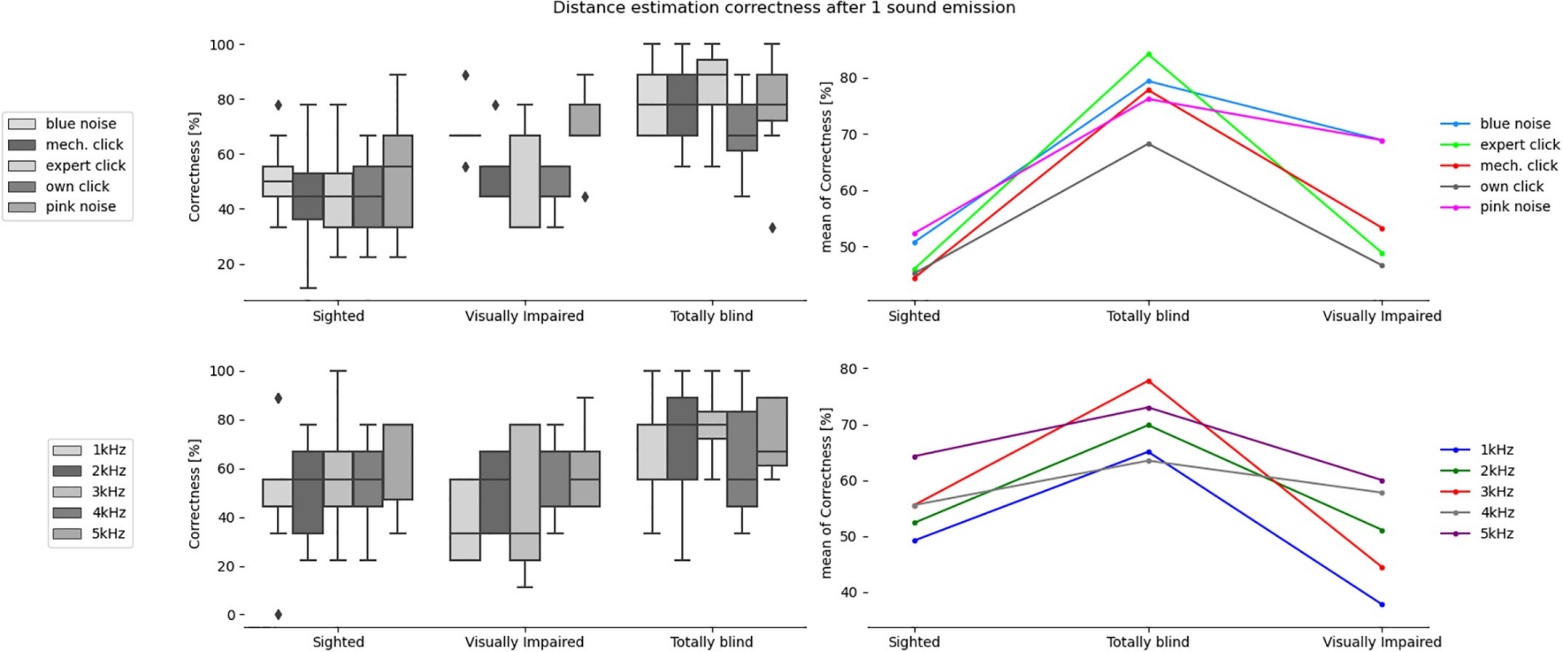
Correctness in estimating distance after a single sound emission for the tested sound types and participants divided into sighted, totally blind and visually impaired groups.

**Fig 6:**
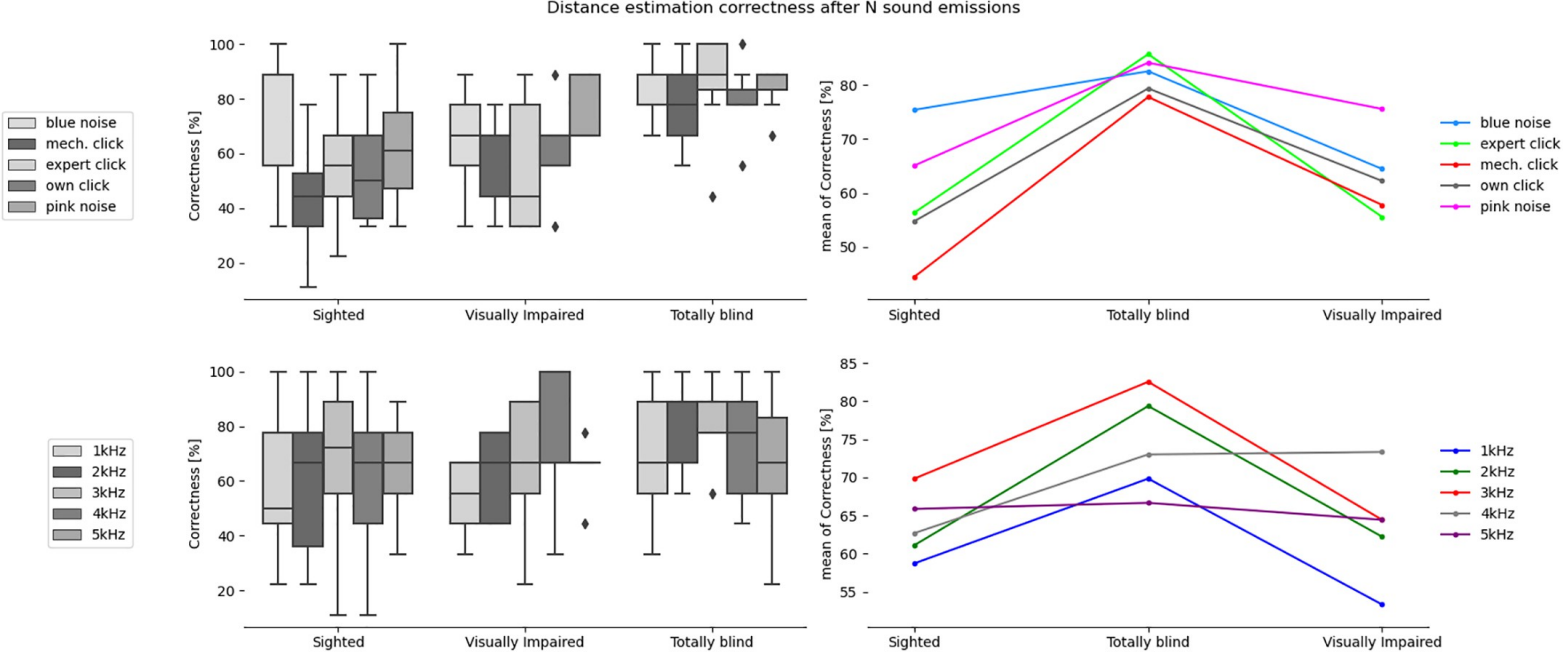
Correctness in estimating distance after N sound emissions for the tested sound types and participants divided into sighted, totally blind and visually impaired groups.

The Pingouin package was used for the ANOVA calculations and SciPy for the Mann-Whitney analysis. The box plots were created using Seaborn. Statsmodels generated Figs 3 to 6, while the remainder of the plots were made in Excel.

The mixed ANOVA (Table 2), where the between-subjects factor was the participants’ group and the within-subjects factor was the sound type, was performed separately for correctness results and certainty ratings obtained after 1 sound emission and after N emissions of a given sound. From the ANOVA results, the interaction effect between the participants groups and the emitted sound type is not statistically significant for all analyzed cases. However, the analysis of each factor separately showed that group type has a statistically significant effect in case of distance estimation accuracy after 1 sound emission. The sound type has statistically significant effect in case of distance estimation accuracy after N sound emissions, as well as in case of direction estimation accuracy and certainty ratings after 1 and N sound emissions. It was also found that the number of sound emissions (N), after which the participant gave the final answer, has a statistically significant effect in case of the interaction between the participant group and the sound type, as well as in case of the sound and participant group itself.

### Differences between groups

**Fig 7:**
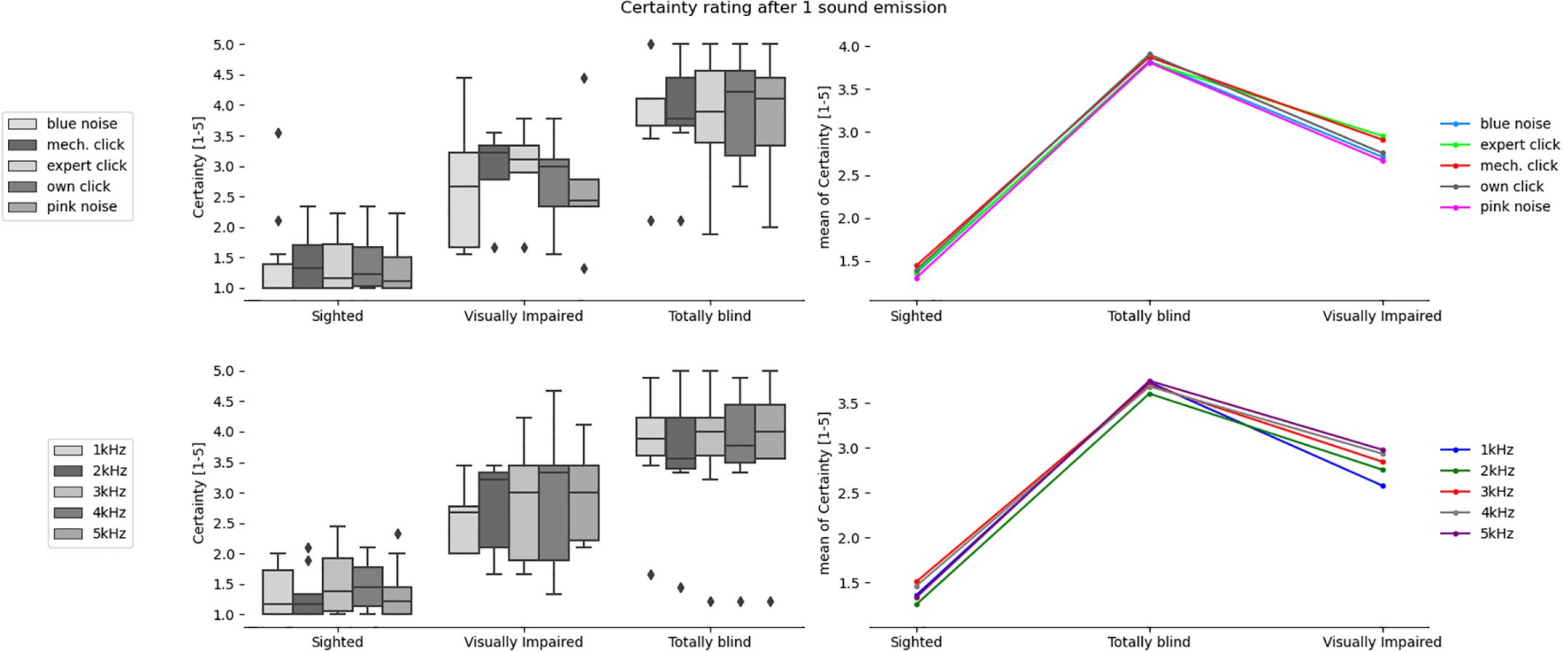
Certainty in the answer after the 1st sound emission for the tested sound types and participants divided into sighted, totally blind and visually impaired groups.

**Fig 8:**
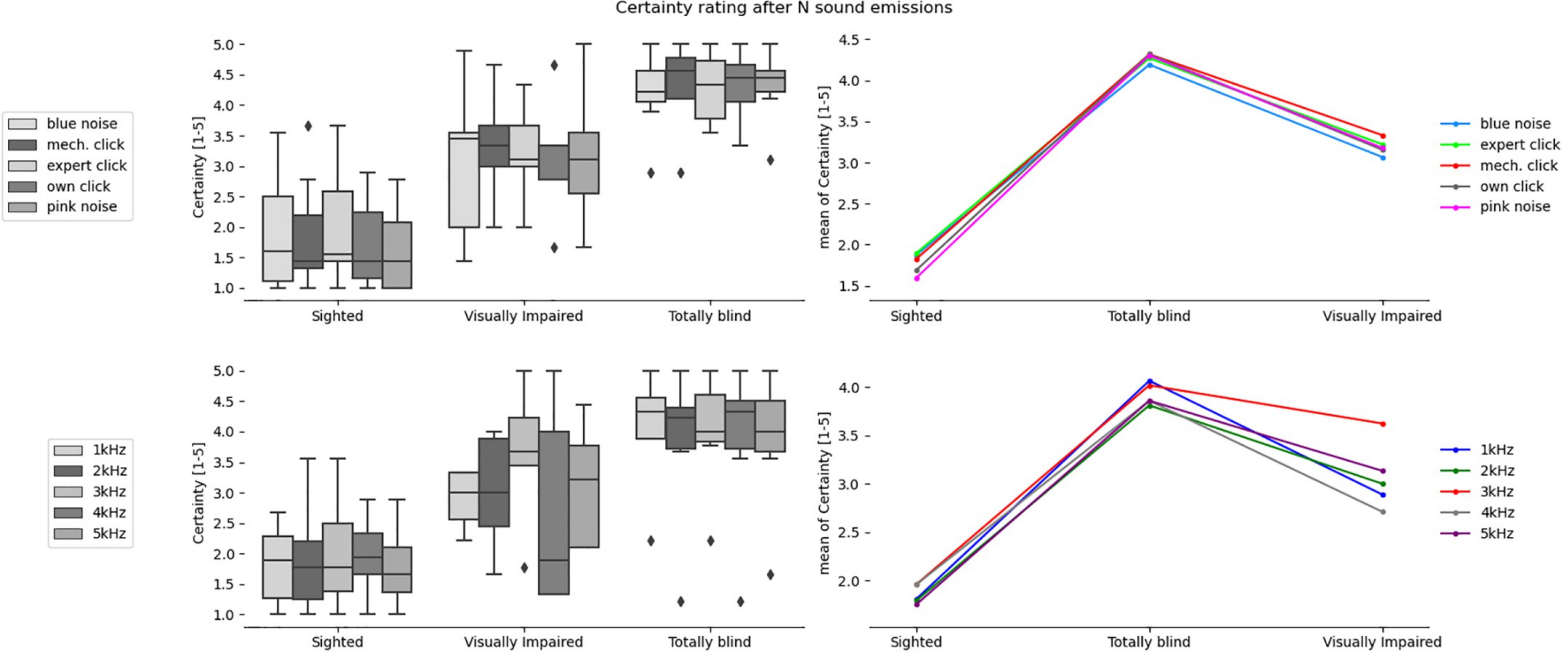
Certainty in the answer after N sound emissions for the tested sound types and participants divided into sighted, totally blind and visually impaired groups.

The results of ANOVA analysis, divided into six sets depending on the type of obstacle location (direction and/or distance) and certainty ratings, for the three participant groups are presented in Table 3 and Figs 3 to 8, including the three parameters: F-value, significance level value α (p-value), F critical value (F crit) and total degrees of freedom. In this part of the statistical analysis the averaged correctness values for all types of sounds were considered, calculated separately for distance and direction. The values for which the significant statistical difference was determined are shown in bold.

**Table 3:** Single ANOVA parameters calculated for pairings of participant groups.

Direction estimation correctness after 1 emission	DF	F-value	α (p-value)	F crit. value
Sighted vs. Totally Blind	**20**	**8.457**	**0.009**	**4.381**
Sighted vs. Visually Impaired	18	0.001	0.978	4.451
Totally Blind vs. Visually Impaired	**11**	**9.857**	**0.011**	**4.965**
**Distance estimation correctness after 1 emission**				
Sighted vs. Totally Blind	**20**	**14.723**	**0.001**	**4.381**
Sighted vs. Visually Impaired	18	0.159	0.695	4.451
Totally Blind vs. Visually Impaired	**11**	**7.525**	**0.021**	**4.964**
**Direction estimation correctness after N emissions**				
Sighted vs. Totally Blind	**20**	**9.299**	**0.006**	**4.381**
Sighted vs. Visually Impaired	18	0.466	0.503	4.451
Totally Blind vs. Visually Impaired	**11**	**11.646**	**0.006**	**4.964**
**Distance estimation correctness after N emissions**				
Sighted vs. Totally Blind	**20**	**7.772**	**0.012**	**4.381**
Sighted vs. Visually Impaired	18	0.181	0.676	4.451
Totally Blind vs. Visually Impaired	11	4.876	0.052	4.964
**Certainty rating after 1 emission**				
Sighted vs. Totally Blind	**20**	**60.576**	**<0.001**	**4.381**
Sighted vs. Visually Impaired	**18**	**7.504**	**<0.001**	**4.451**
Totally Blind vs. Visually Impaired	11	2.965	0.116	4.965
**Certainty rating after N emissions**				
Sighted vs. Totally Blind	**20**	**59.025**	**<0.001**	**4.381**
Sighted vs. Visually Impaired	**18**	**13.348**	**0.002**	**4.451**
Totally Blind vs. Visually Impaired	11	3.690	0.084	4.965

It can be observed that in all cases in Table 3 there is a significant difference between the group of sighted participants and totally blind participants, but not the visually impaired participants. In 3 out of 4 analyzed cases related to obstacle location there exists statistically significant difference between totally blind and visually impaired persons. In case of certainty ratings the significant statistical difference can be observed also for sighted and visually impaired participants. On the other hand, the differences are never significant for sighted and visually impaired groups in case of obstacle localization. The implication is that legally blind persons who can still rely on rudimentary light sensitivity are not likely to develop echolocation skills significantly greater than an average sighted person.

**Fig 9:**
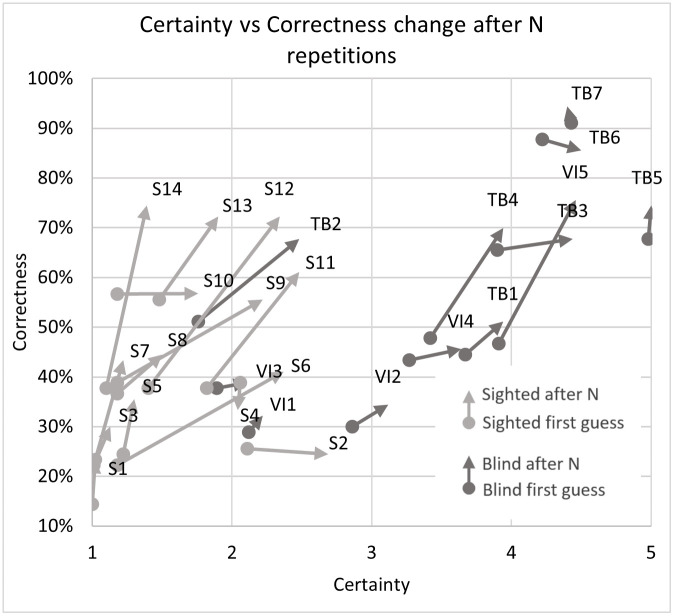
Correctness and certainty change after N signal repetitions for all 26 participants.

The most clear division between the sighted and blind participants was the very large difference in certainty, which allows a clear separation of groups (if the average certainty was above 3 –the participant or a subgroup was almost certainly not sighted). This can be most clearly seen in Figs 7 and 8 illustrating the differences between the means, as well as in Figs 9 and 13 where certainty was used as the horizontal axis. The sighted participants also made, on average, larger improvements between the first guess and the answer given after N repetitions of the signal, which will be discussed in the next section.

**Fig 10:**
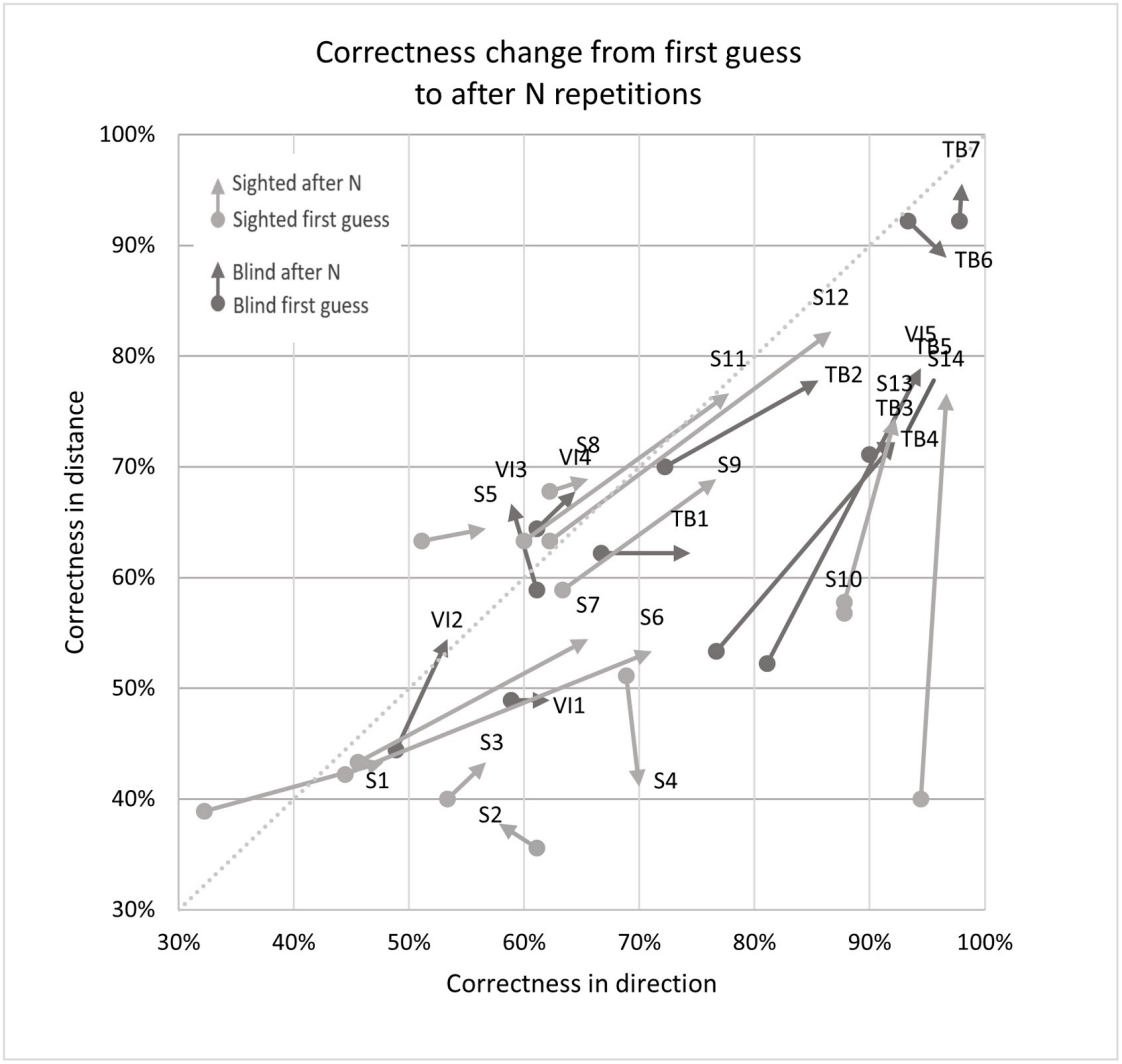
Correctness change in distance and direction after N signal repetitions for all 26 participants.

**Fig 11:**
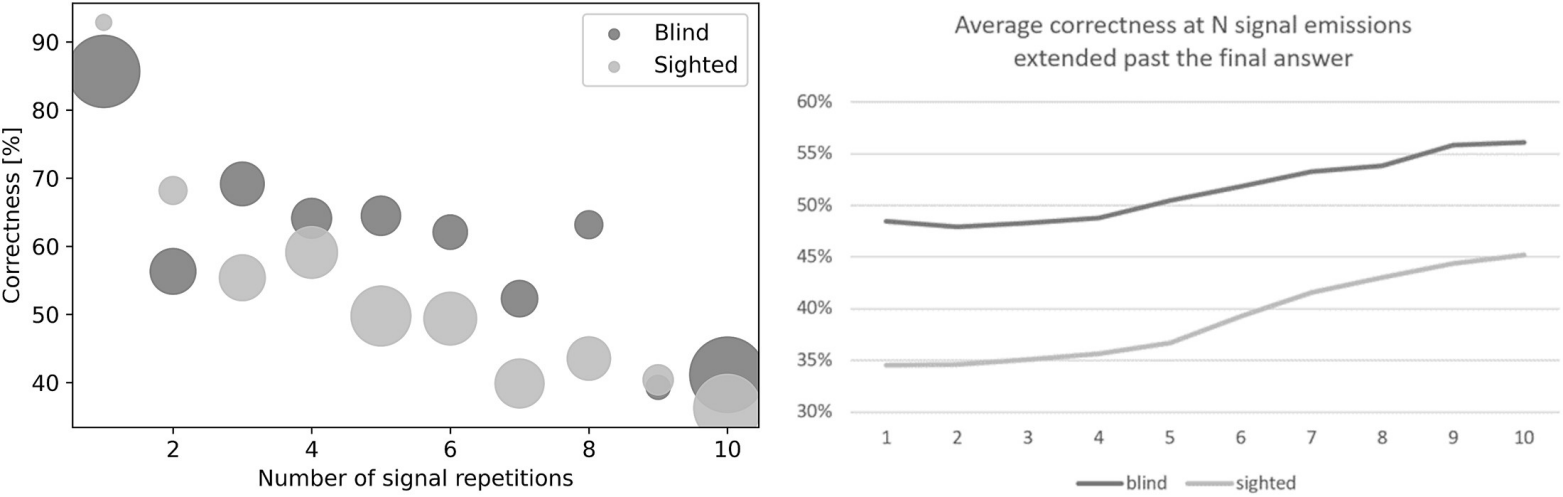
Left—Average correctness for participants that needed N signal repetitions to provide their final answer. Area of the circles corresponds to the number of participants that stopped at the given N. Largest circle area corresponds to 12 participants. **Right—Average correctness at the Nth repetition after each participant’s final answer was extended to all further repetitions.**

**Fig 12:**
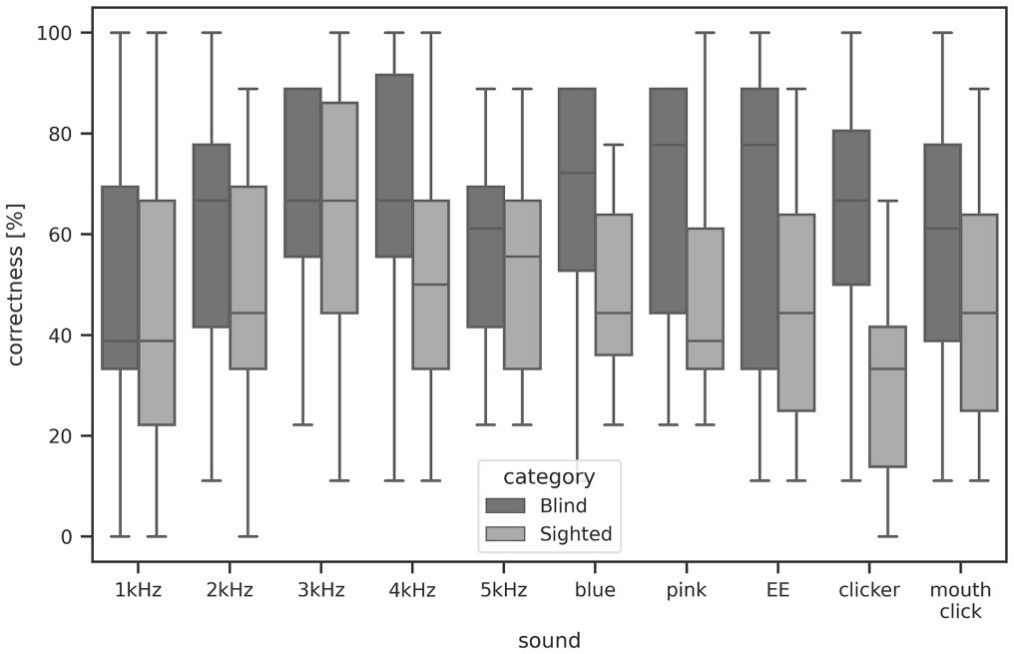
Quartile distribution plots for the ten compared sounds.

**Fig 13:**
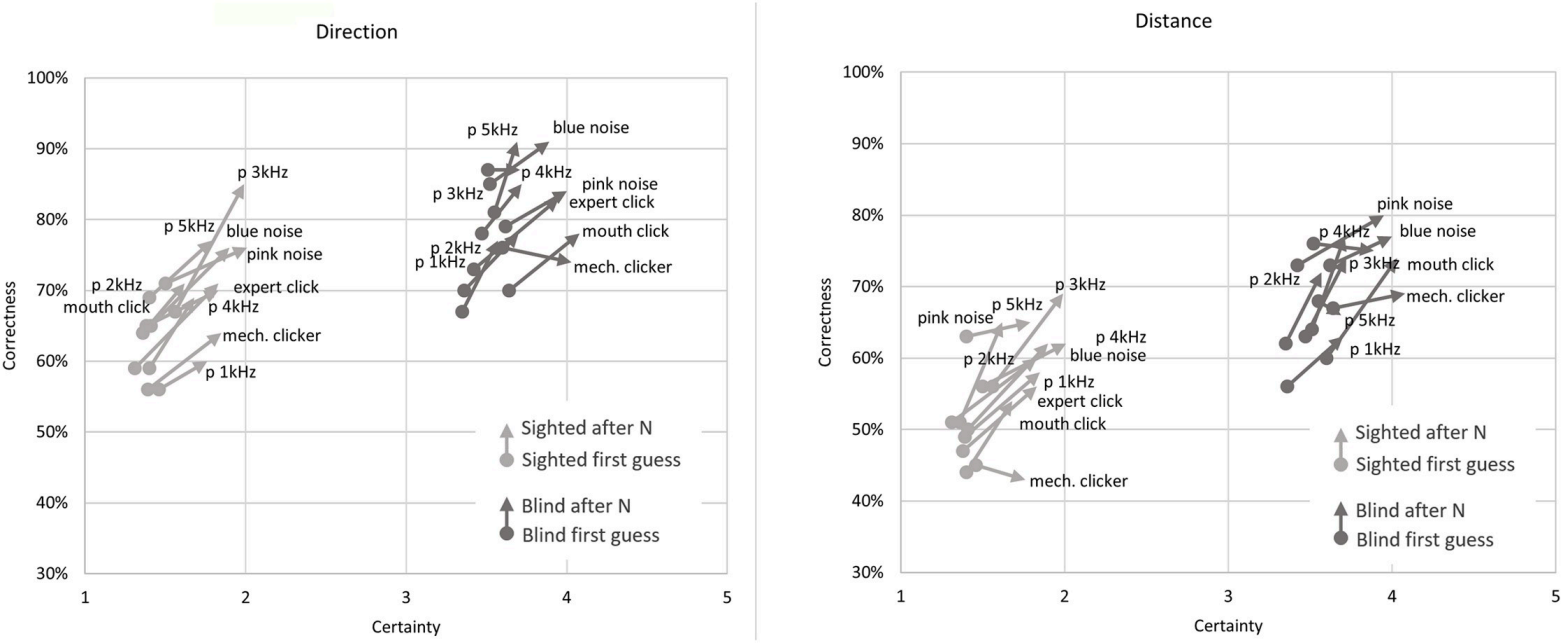
Comparison of correctness in direction and distance estimates for the 10 different sound sources.

### Changes in correctness after additional signal repetitions

The volunteers could choose to repeat the emission of a sound signal from 1 to 10 times, until they either felt confident in their first answer or decided to change the answer to a different location.

As seen in Figs 8 and 15 the change in correctness between the first answer and after listening to the echo N times is greatest for sighted volunteers and interestingly the smallest for the totally blind (especially the experienced experts TB5 and TB7) as well as the visually impaired but inexperienced echolocators. The most likely explanation is that the best echolocators did not need the additional repetitions as their answers were already correct, while the worst echolocators did not gain a significant improvement from additional repetitions, which can be observed in Figs 9 and 10. In addition from these figures we can see the change in correctness was considerably larger than the change in certainty as well as the change in the correctness of identifying distance greater than in direction.

**Fig 14:**
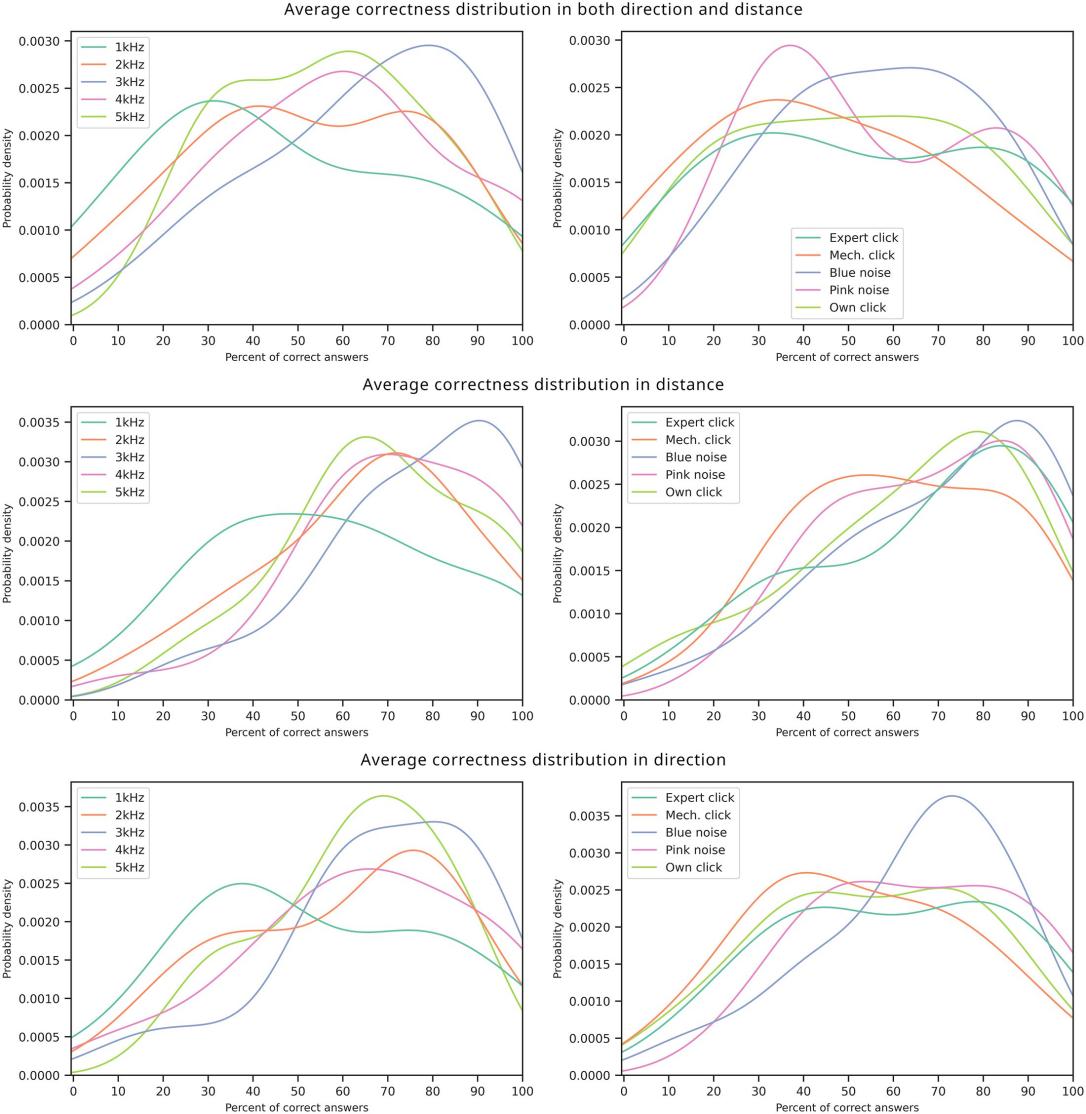
Distribution of participants with the given percentage of correct answers for each sound after smoothing the results with a kernel density estimating function (KDE) for percussive sounds (left) and other sounds (right), in three rows—Overall correctness, distance correctness only, and direction correctness only.

**Fig 15:**
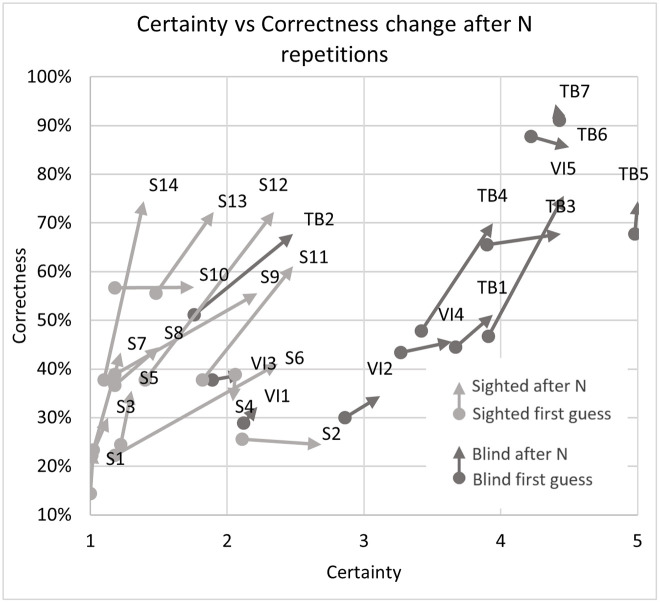
Correctness change after N signal repetitions for all 26 participants.

In Fig 9 (and later Fig 12) we can clearly observe a grouping of vectors for sighted (light grey) and blind (dark grey) participants and higher correctness rates for direction estimation. The change in correctness was generally higher for sighted persons (light grey color). It is also evident that more people were correct with direction on the first guess, but additional guesses improved the distance assessments.

Fig 11 shows the dependence of correctness of responses as a function of the number of signal repetitions (number of times a signal was listened to) during the echolocation tests. Data were determined separately for blind and sighted subjects and normalized to the number of test subjects. The radius of the marker depends on the number of records for a given number of signal repetitions. It can be noticed that blind people were significantly more confident than sighted people after the echolocation sound was played or generated once. Both user groups, when in doubt, mostly used N = 10 sounds. One surprising irregularity was that the small group of answers where sighted participants finished after only 1 additional signal repetition was on average more frequently correct than the blind group.

Although there was an apparent trend that additional repetitions were associated with lower correctness (Fig 11 left), it might have been caused by participants correctly stopping the additional repetitions when they felt confident in their answers. In order to observe whether additional signal emissions actually led to improvements in correctness we extended the participants’ final answers to further repetitions, e.g. if a participant stopped at N = 3, we simply kept their answer for all further Ns (4–10). This allowed to measure the average correctness at all “N” and observe a clear trend (Fig 11 right), which was also statistically confirmed (F = 71.75, α = <0.001).

### Differences between sounds

The main goal of the study was to determine whether some of the chosen sounds had a significant effect on the correctness of echolocation. Due to the large variations in the participant’s echolocation skills, it was difficult to find a sound with a clear advantage, however significant differences were observed for some sound pairs.

All types of tested sounds were compared in pairs and for all cases of average correctness values: distance after 1 click, distance after N clicks, direction after 1 click, direction after N clicks, mean value of correctness after 1 and N clicks. The Mann-Whitney U Test was used, as it was assumed that the samples are not normally distributed. The significance level value α was set to 0.05. Tables 4–8 present the Mann-Whitney U Test parameters calculated for pairs of sounds where the set difference was found to be statistically significant. There were five types of sounds that were found to be statistically better from some of the others: 3kHz (Table 4), 4kHz (Table 5) and 5kHz percussion (Table 6), as well as blue (Table 7) and pink noise (Table 8). Percussion sounds were found to give better average obstacle detection correctness in case of distance and direction. The blue noise and pink noise sounds sources appeared to be better at determining distance to an obstacle. The quartilion distribution plots for the ten compared sounds are shown in Fig 12, with a division into sighted and blind groups.

**Table 4:** Mann-Whitney U Test results for blue noise.

Statistically better sound: Blue noise			
When compared to	Significant difference for	Correctness difference	p-value
1kHz percussion	distance and direction;	18.38%	0.039
	all participant groups;		
	1st sound		
Mechanical clicker	distance and direction;	14.43%	0.036
	all participant groups;		
	N repetitions		
Mouth click/Hand clap	distance;	13.00%	0.039
	all participant groups;		
	1 sound		

**Table 5:** Mann-Whitney U Test results for pink noise.

Statistically better sound: Pink noise			
When compared to	Significant difference for	Correctness difference	p-value
1kHz percussion	distance;	18.52%	0.046
	blind participans;		
	1st and N repetitions		
2kHz percussion	distance and direction;	11.11%	0.007
	all participant groups;		
	1st sound		
5kHz percussion	distance;	14.82%	0.036
	blind participants;		
	1st sound		
Mechanical clicker	distance and direction;	16.96%	0.039
	all participant groups;		
	1st and N repetitions		

**Table 6:** Mann-Whitney U Test results for 3kHz percussion sound.

*Statistically better sound*: *3kHz percussion*			
When compared to	Significant difference for	Correctness difference	p-value
1kHz percussion	distance and direction;	17.56%	0.025
	all participant groups;		
	N repetitions		
Mechanical clicker	distance and direction;	22.38%	0.012
	all participant groups;		
	N repetitions		
Mouth click/Hand clap	distance and direction;	14.47%	0.049
	all participant groups;		
	N repetitions		
Pink noise	direction;	15.08%	0.046
	sighted participants;		
	N repetitions		

**Table 7:** Mann-Whitney U Test results for 4kHz percussion sound.

*Reference sound*: *4kHz percussion*			
Sound	Influence type	Correctness difference	p-value
1kHz percussion	distance and direction;	15.24%	0.046
	all participant groups;		
	1st sound		
2kHz percussion	distance and direction;	16.33%	0.023
	all participant groups;		
	1st sound		
Mechanical clicker	distance and direction;	15.98%	0.049
	all participant groups;		
	1st and N repetitions		

**Table 8:** Mann-Whitney U Test results for 5kHz percussion sound.

*Reference sound*: *5kHz percussion*			
Sound	Influence type	Correctness difference	p-value
1kHz percussion	distance and direction;	12.82%	0.039
	all participant groups;		
	1st and N repetitions		
2kHz percussion	distance and direction;	11.54%	0.048
	all participant groups;		
	1st sound		
Blue noise	distance;	13.49%	0.034
	sighted participants;		
	1st sound		
Mechanical clicker	distance and direction;	18.35%	0.035
	all participant groups;		
	1st and N repetitions		
Expert click	distance;	18.26%	0.012
	sighted participants;		
	1st sound		

The box plots in Fig 12 show the average correctness for different sounds separately for sighted and blind participants, while separate box blots for the separate groups and localizations were shown earlier in Figs 3 to 8. From the test set, the best performing sounds were those with dominant frequencies at 3-4kHz and pink and blue noise, but the advantage is significant only over the worst sounds (the mechanical clicker and the own sound).

A separate analysis was attempted to compare the sounds using a probability density plot—extrapolating from the gathered dataset to answer how likely it was to get a certain average number of correct answers with the given sound? These distribution plots are shown in Fig 14 which for clarity was divided into percussive sounds and other sounds.

**Fig 16:**
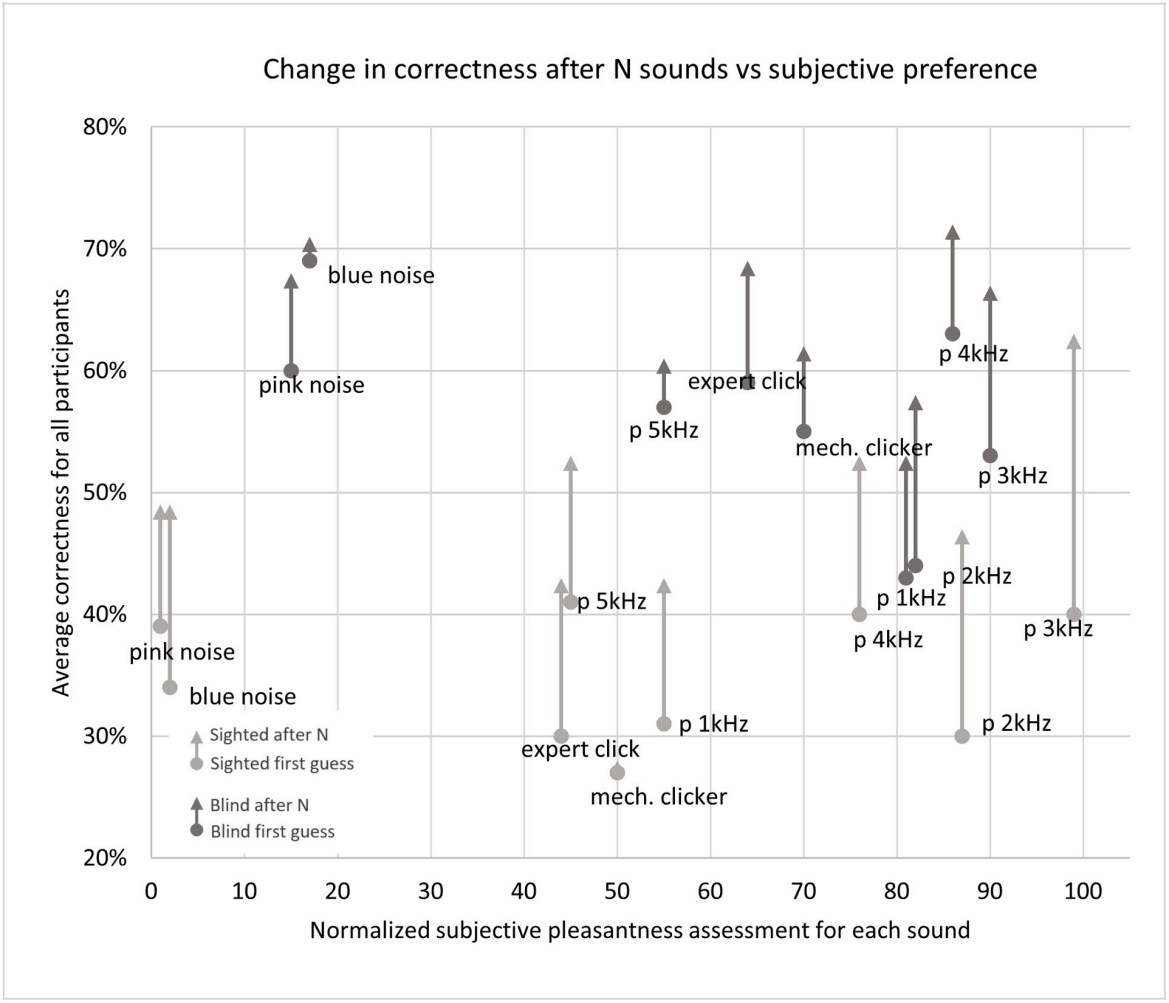
Change in correctness for the 9 tested sounds vs the sounds’ subjective aesthetic ranking. The participant’s own sound was excluded from this ranking.

The sounds were also aesthetically assessed by all test participants in a post-test survey. The sounds were ranked from best to worst (without the participant’s own sound), with 9 points for the best sound and 0 for the worst. Afterwards, the score was summed and normalized to 100 relative to the highest obtained score. As seen in Fig 16 the most preferred sound among both the blind and sighted participants was the 3kHz percussion generated with the Risset drum algorithm, while the highly effective blue and pink noise were ranked as the least pleasant.

## 4. Discussion

The statistical analysis of the results was conducted using two methods: mixed and one-way ANOVA for comparing different groups of participants, and the Mann-Whitney U test for comparing different sound types. The analysis showed that the group of totally blind participants demonstrated significantly better overall echolocation skills than the groups of visually impaired and sighted participants, which was an expected outcome. However, there was no statistically significant difference between the legally blind with residual vision and participants with healthy eyesight. None of the test participants had formal training in echolocation, although the top blind echolocators did explain that they had learned it implicitly through prolonged experience. The conclusion may be that as long as visual impairment is not total, residual eyesight along with a white cane are primarily used for orientation and mobility, without the need for developing any echolocation skills. What the visually impaired and the totally blind groups had in common was a significantly higher certainty in their answers. This can be explained by either having no way to easily verify the correctness of their assumptions about their echolocation skills, or by the participants trying to meet the expectations of the researchers.

In the case of sound comparisons, it was found that both types of noise, blue and pink, were significantly better than low-frequency percussion sounds and mechanical clickers for all groups of participants. In the case of blue noise, distance estimation was especially improved. Percussion sounds of 3kHz, 4kHz, and 5kHz turned out to be the best types of sounds for direction estimation. The 3kHz percussion showed better results after N repetitions, while 4kHz and 5kHz percussion gave good detection rates both after 1 and N repetitions. The two sounds that were statistically worst were the mechanical clicker and 1kHz percussion.

The results of the statistical analysis suggest that sighted participants performed best with 3kHz and 4kHz percussion sounds, while the blind group was most frequently correct with blue and pink noise. However, the subjective ratings of sounds by all participants ranked the noises as the least pleasant to use, leading to the conclusion that 3kHz sounds should be the preferred choice. These results are in line with previous studies and theory [3]. The best sounds have a frequency peak in the range that the human ear is most sensitive to, i.e. 3kHz, as well as sufficiently wide spectra (noises). The worst-performing sounds had less high frequency content, a lower frequency peak, and/or were less consistently repeatable.

The study also drew attention to a number of issues regarding the design of future echolocation tests. The first one is that the participants cannot be simply divided only into just two categories—blind and sighted, as there is a significant difference in echolocation ability between totally and partially blind persons. A lot of studies did add a separate category of “expert echolocators” [17], but we believe the division by level of visual impairment may be a better one, though it will require to conduct tests with a larger number of participants. Another is the issue of how time consuming the real-life tests are in comparison to recordings. Some streamlining of the procedures and automation of the testing process may be in order, such as the Echobot which can mechanically move the echolocated obstacle [23].

## 5. Conclusions

A number of conclusions can be drawn from the presented trials. Firstly, it is clear that nearly all humans can echolocate significantly above random, even complete novices with healthy eyesight. Out of the 26 study participants only two had average correctness levels that were at chance level. The highest correctness in obstacle localization was achieved by experienced totally blind testers, which was no surprise. However, the visually impaired participants that were only legally blind and were used to relying on some level of residual light sensitivity performed on average the same as completely novice participants with healthy eyesight. The conclusion is that the totally blind learn echolocation primarily through forced experience, but there is nothing preventing other visually impaired and even normally sighted persons from learning this skill through specialized training, such as proposed in [4] or [2].

It was difficult to find “the” optimal sound for human echolocation, but the results confirm that sounds with a centre frequency near 3-4kHz and a sufficiently wide spectrum provide higher correctness rates than those with lower frequency peaks. Blue noise, which has higher spectral content, was marginally better than pink noise, which has more energy in the lower frequencies. This was especially evident in the perception of distance.

It was also clear that the sounds that were the least repeatable, i.e. had variations due to being generated by the participants and not replayed via a speaker, were significantly worst in the tested set.

The results may be useful for developing training curricula, e.g. teaching future echolocators to generate sounds that are relatively high in frequency (3-4kHz) and wide in spectrum. Some blind echolocators may benefit from using portable loudspeakers to continuously generate a perfectly repeatable source sound, a practice which two of the trial’s best echolocators have started to experiment with in everyday life.
